# Enzymatically Synthesized Poly(Gallic Acid) Modulates Methionine Synthase Activity and Neuroblastoma Morphology in Contrast to Phthalate‐Type Endocrine Disruptors

**DOI:** 10.1002/bip.70074

**Published:** 2025-12-11

**Authors:** Gabriela García‐Cerón, Iván H. Bello‐Cortés, Yazmín Ramiro‐Cortés, Manuel Gutiérrez‐Aguilar, Marcos Rosetti Sciutto, Francisco Sánchez‐Bartéz, Miquel Gimeno, Roeb García‐Arrazola

**Affiliations:** ^1^ Depto. de Alimentos y Biotecnología, Facultad de Química Universidad Nacional Autónoma de México (UNAM) Ciudad de México México; ^2^ Posgrado en Ciencias Biológicas Universidad Nacional Autónoma de México Ciudad de México México; ^3^ Instituto de Fisiología Celular Universidad Nacional Autónoma de México (UNAM) Ciudad de México México; ^4^ Depto. De Bioquímica, Facultad de Química Universidad Nacional Autónoma de México (UNAM) Ciudad de México México; ^5^ Instituto de Investigaciones Biomédicas Universidad Nacional Autónoma de México (UNAM) Ciudad de México México; ^6^ Unidad de Investigación Preclínica (UNIPREC), Facultad de Química Universidad Nacional Autónoma de México, Ciudad Universitaria Ciudad de México México

**Keywords:** endocrine‐disrupting compounds, methionine synthase activity, redox‐regulating molecules

## Abstract

The effect of the enzyme‐mediated poly(gallic acid) (PGAL) as a potential redox regulator or redox activity compound (RAC) on the morphology of human neuroblastoma SH‐SY5Y cells and its methionine synthase (MS) activities is contrasted to those for disrupting compounds (EDC). For that, we study the effects of di(2‐ethylhexyl)phthalate (DEHP) and monobutylphthalate (MBP) as common EDCs. The results show the expected significant decrease in cell density and predominance of phenotype N associated with shorter neurites after exposure to EDCs; however, homogeneous cell density and an S phenotype consistent with the control are observed after exposure to the polymeric RAC, and compared to other reported RAC metabolites, sulforaphane (SFN) and its precursor gallic acid (GA). Regarding the enzymatic activity of MS, a 64% increase is observed in the presence of EDCs. Surprisingly, control GA also shows a 35% increase in MS enzymatic activity, but this stable multiradical polyanion derivative has an average decrease of 51%. To the best of our knowledge, this is the first time that MS enzymatic activities‐to‐risk of endocrine disruption relationships, compared to that of a polymeric RAC, have been established using neuroblastoma cell cultures, laying groundwork for future research in neurobiology and environmental health.

## Graphical abstract

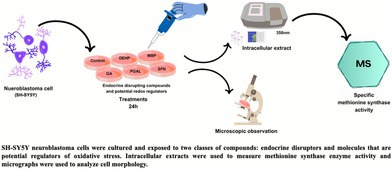

## 1 Introduction

Emerging contaminants encompass a diverse array of substances with varying origins and chemical properties, many of which extend beyond endocrine disruption to impact overall human and environmental health [1]. One category of emerging contaminants is substances or exogenous mixtures composed of endocrine‐disrupting compounds (EDCs), whose exposure to humans is mainly by ingestion [2, 3]. Chemical compounds such as phthalates, bisphenol‐A (BPA), polybrominated diphenyl ethers, nonylphenols, and natural and synthetic estrogens, such as genistein and daidzein [4] are among the main EDCs present in foods. These emerging EDCs could be a key factor in identifying the causes of various metabolic conditions such as diabetes, cancer, congenital malformations, cardiovascular diseases, reproductive system disease, immune system disorders, and neurodevelopmental issues [5, 6]. Although regulations limit the concentrations of these substances in food and mitigate their potential negative effects on human health [7, 8], there is an urgent need for proposals to revise and lower maximum daily exposures [2]. The established regulatory limits for phthalates are directly supported by evidence demonstrating their neurotoxic effects on child development [9, 10].

Phthalates have been recognized as the main source of involuntary exposure to endocrine disruptors in humans via aliments [11]. Among those, DEHP and MBP have been reported to diminish cell viability [12] resulting in neuronal death [13] due to and toxicity and apoptotic effects [14]. Many results conclude that an imbalance between prooxidant and antioxidant molecules is associated with damage to essential cellular processes in the early development stages of the central nervous system (CNS), which is crucial for the explanation of several neural disorders [15]. In this regard, this redox imbalance can be regulated by endogenous and exogenous antioxidants that neutralize reactive oxygen species (ROS) and related oxidative enzymes [16]. A redox DNA methylation mechanism has been proposed, suggesting an MS‐dependent metabolic relationship between oxidative stress and methylation, which may impact gene regulation processes in genetically vulnerable individuals [17]. In this regard, the methionine synthase (MS) activity is essential for homocysteine‐dependent methylation stimulated by the dopamine D4 receptor, the unique signaling process that promotes the synchronization of neural networks [18]. Therefore, dysregulation of MS activity can lead to alterations in DNA methylation patterns; consequently, neurotransmitter production leads to changes in behavior and overall cognition. Thus, increasing MS activity can positively impact DNA methylation [19]. Conversely, its inhibition is associated with global DNA hypomethylation, thus promoting the expression of sensitive genes that are key in epigenetic mechanisms [20]. MS is also necessary for folate‐dependent methylation from phospholipids in the membrane throughout the dopamine D4 receptor [18]. Therefore, MS activity could play an important role in the neuronal plasticity/architecture generated by neurotransmitters [21]. The ability of IGF‐1 (insulin‐like growth factor‐1) and dopamine to increase MS activity in SH‐SY5Y neuroblastoma cells has been demonstrated through a mechanism regulated by PI3‐kinase and MAP‐kinase pathways [19]. This identifies MS activity as a determinant in homocysteine and S‐adenosylhomocysteine (SAH) levels, which in turn influences the methylation index (S‐adenosylmethionine (SAM) /SAH Ratio). Consequently, cellular REDOX imbalance is associated with the endocrine‐disrupting effect related to MS activity.

It is worth noting that the SH‐SY5Y cell line is a widely used in vitro research model due to its neuronal function and differentiation specifics [22]. Particularly, this human cell line has been studied in neurodevelopmental disorders and neurodegenerative conditions due to its adrenergic and dopaminergic characteristics [23]. Additionally, several studies associate the exposure to EDC with epigenetic modifications, including DNA methylation. For example, exposure to BPA has been shown to trigger DNA and histone methylation in SH‐SY5Y cells [24].

This explains our interest in vitro exposure of SH‐SY5Y neuroblastoma cells to emerging contaminants such as phthalate‐type EDCs and RACs to assess the influence on MS enzymatic activity, since this enzyme is key to sensor cellular oxidation and cell morphologies. Polygalic acid has been shown to have an antioxidant effect on epithelial cells through its quantification by DPPH [25]. This polymer is enzymatically produced via the polyoxidation of gallic acid (GA) catalyzed by laccase from *Trametes versicolor*, a medicinal fungus also named Turkey tail, offering a non‐cytotoxic thermo and photo‐stable multi‐radical polyanion [26]. Specifically, GA has been reported for its antitumor activity related to its REDOX regulatory effect [27] The results are compared to sulforaphane (SFN) as a natural radical suppressor agent [26, 28], as well as its monomer precursor GA. SFN has been ranked as one of the 40 most promising molecules by the National Cancer Association in the USA [29] and in a systematic review, it was rated as one of the substances with more potential benefits in treatments for autism [30, 31].

The scope of the present study is to evaluate the neuroprotective effect of molecules known for their antioxidant effect such as polygalic acid (PGAL), GA, and sulforaphane (SFN). This work contributes to ascertaining the detrimental effect of EDCs, and that for PGAL, toward potential routes to preserve SH‐SY5Y cells from these harmful chemicals.

## 2 Materials and Methods

### 2.1 Materials

SH‐SY5Y neuroblastoma cell line was obtained from ATCC (CRL‐2266). Gibco Dulbecco's Modified Eagle Medium (DMEM) was acquired from ATCC (30‐2003), as well as F12K Medium (Kaighn's Modification of Ham's F‐12 Medium) ATCC (30‐2004). The Cell Proliferation Kit II (XTT) (11465015001) was acquired from Roche. Fetal Bovine Serum (FBS) was obtained from ATCC (30‐2020). Dithiothreitol (DTT), L‐homocysteine, S‐Adenosyl Methionine (AdoMet), Hydroxocobalamin, and Methyl‐tetrahydrofolate (CH3THF) were purchased from Sigma Aldrich. PGAL was enzymatically synthesized by laccase from *Trametes versicolor* (Sigma Aldrich; specific activity of 0.31 U/mg/min) in buffer solution following a previously reported procedure [25]. The *M*_n_ and PDI for PGAL, 5,644 Da and 1.94, respectively, were determined by SEC using an HPLC Agilent (USA), equipped with a Refractive Index Detector using two Ultrahydrogel 500 (7.8 × 300 mm) columns in series (Waters, USA) placed in a thermostat (30°C) and calibrated with polyethylene glycol standards. Samples were eluted with ultrapure deionized water (Simplicity UV Millipore, USA) with LiCl (0.1 M) in a 0.6 mL/min flow. All samples were dissolved in the mobile phase and filtered (0.45 μm) prior to injection in the chromatographer. All other reagents were analytical grade and commercially available.

### 2.2 Cell Culture

Cell cultures were routinely passaged and maintained as recommended by ATCC, in the Unidad de Investigación Preclínica (UNIPREC), of the Faculty of Chemistry. SH‐SY5Y neuroblastoma cells were seeded using DMEM/F12 medium supplemented with 10% FBS. The cells were incubated at 37°C in an atmosphere of 5% CO_2_. Cell propagation was considered depending on the experiment. Cytotoxicity was evaluated in 96‐well plates with ~16,000 cells per well, and for Methionine Synthase Enzymatic Activity (lysate extraction), 6‐well plates with ~150,000 cells were used. Exposure to substrates was performed after 5 days of culture, when SH‐SY5Y cells reached a subconfluent state (~70%–80%) of growth based on experimental results as reported elsewhere [23, 32].

### 2.3 Exposure to Different Substrates (Variety of EDCs)

Exposure to DEHP and MBP was conducted 5 days after cell propagation. Different concentrations dissolved in DMSO ≤ 1% v/v were applied: 1, 10, 25, 50 μM for both cases, and each condition was tested in technical triplicate, using three wells per concentration within the same plate, all treated under identical conditions. Cells were incubated at 37°C in an atmosphere of 5% CO_2_ for a period of 24 h. The concentrations used in our study were established based on previous studies on phthalates [13], which investigated the cytotoxic effect of DEHP in SH‐SY5Y neuroblastoma cells.

The choice of DMSO was based on its high compatibility with neuronal cultures and the need to ensure stable experimental conditions during EDC exposure. DMSO is widely used as a vehicle in SH‐SY5Y cells due to its ability to solubilize hydrophobic compounds and its low impact on membrane integrity and cellular metabolism when used at concentrations ≤ 1% v/v. Although alternative solvents such as ethanol or corn oil are more efficient for DEHP solubilization, their use in human neuronal cultures has been associated with alterations in membrane permeability, morphological modifications, and undesirable effects on intracellular signaling [33]. Therefore, DMSO was used under controlled conditions in accordance with methodologies reported elsewhere using SH‐SY5Y for phthalate exposure in vitro studies [13, 22].

### 2.4 Exposure to Different Redox‐Regulating Molecules

Exposure to RAC was carried out 5 days after cell propagation. Different concentrations were applied: GA 0.5, 0.75, 1, 1.5 μg/mL; PGAL 0.5, 1, 5, 10 μg/mL; SFN 1, 1.5, 2.5, 5 μM. Each condition was tested in technical triplicate, using three wells per concentration within the same plate, all treated under identical conditions. Cells were incubated at 37°C in an atmosphere of 5% CO_2_ for a period of 24 h. For the redox regulators evaluated in this study, we considered concentrations previously reported in studies assessing the oxidative stress attenuation effect of GA in SH‐SY5Y neuroblastoma cells [34]; the impact of PGAL on proliferation and adhesion in THP‐1, fibroblasts, HCT 116, and HT‐29 cells [26] and the prevention of mitochondrial impairment and neuroinflammation by SFN in SH‐SY5Y neuroblastoma cells and mouse BV2 microglial cells [35]. DMSO was used as the vehicle for SFN, since it is an appropriate solvent for hydrophobic compounds and widely accepted in SH‐SY5Y cultures. The final concentration was maintained ≤ 1% v/v, in agreement with protocols validated in previous studies [13, 36, 37, 38, 39]. In contrast, GA and PGAL, highly water‐soluble compounds, were dissolved directly in DMEM/F12, avoiding organic solvents and guaranteeing stability and compatibility with the cell system [34, 40].

The use of specific vehicles for each substance made it possible to maintain experimental integrity and to ensure that the effects observed were attributable exclusively to the treatments evaluated.

### 2.5 Cytotoxicity

To evaluate different concentrations of EDCs and redox‐regulating molecules, a cytotoxicity assay was performed using the Cell Proliferation Kit II (Merck). The culture medium was removed, and 1 μL of the electron coupling reagent and 50 μL of XTT reagent were added directly to each well. The plate was incubated at 37°C in an atmosphere of 5% CO_2_ for 4 h. Finally, the samples were measured in an Epoch spectrophotometer at 490 nm.

### 2.6 Cell Lysis and Protein Quantification

Lysates were obtained by mechanical scraping. First, the culture medium was removed, and the cells were washed once with 1 mL of 1X PBS. Then, 200 μL per well of lysis solution (RIPA buffer) was added for 10 min for scraping. The extract was collected in Eppendorf tubes and centrifuged for 5 min to recover the supernatant. Subsequently, the protein content of each lysate was measured using the Bradford method.

### 2.7 Enzymatic Methionine Synthase Assay

For MS activity, the protocol described in a previous study was adapted for a total assay volume of 200 μL in 96‐well plates [41]. The volume of each lysate was added to standardize to 0.025 mg of protein in the assay. The content was adjusted with Milli‐Q water to a final volume of 98.4 μL. Then, 16 μL of 1.0 M KPO_4_ buffer (pH 7.2), 8 μL of 500 mM DTT, 0.8 μL of 3.8 mM S‐adenosylmethionine, 0.8 μL of 100 mM L‐homocysteine, and 16 μL of 500 μM hydroxocobalamin were added. A pre‐incubation was performed at 37°C in a 5% CO_2_ atmosphere for 5 min. Subsequently, the reaction was initiated by adding 20 μL of 4.2 mM methyl‐THF, mixed gently, and incubated for 10 min at 37°C in a 5% CO_2_ atmosphere. The reaction was stopped by adding 40 μL of 5 N HCl containing 60% formic acid, followed by incubation at 80°C for 10 min. Finally, the plate was cooled to room temperature and analyzed using the Epoch microplate reader at 350 nm.

### 2.8 In Silico Docking Prediction

The molecular docking studies between DEHP, MBP, GA, and SFN in human MS (PDB: 4CCZ) were performed with AutoDock Vina using Chimera as previously described [42]. For PGAL, an oligomer of eight gallic acid units (*n* = 8, ~1.4 kDa) was employed as a representative model. The full‐length polymer (*n* ≈ 32, ~5 kDa) exceeded the practical size limits of AutoDock Vina; consequently, the 8‐unit construct was chosen as a computationally tractable alternative that retains the key functional groups, steric features, and chemical properties relevant for binding.

### 2.9 Image Analysis

We analyzed the changes in SH‐SY5Y neuroblastoma cell morphology according to the treatment used. For each well, three non‐overlapping regions were randomly selected, yielding an *n* = 25 per treatment in order to ensure consistent cell density. These regions were analyzed under identical magnification and exposure settings. Image processing and morphometric quantification were performed in ImageJ using micrometer measurements [43] and the resulting data were subsequently used for statistical analysis. Cells with well‐defined borders were selected, and the visibly longer ends of both the soma and neurites were measured to identify their lengths.

### 2.10 Statistical Analysis

Data were analyzed on RStudio using two‐way ANOVA followed by Tukey's post hoc test. Values were expressed as mean ± standard deviation (SD). Differences were considered significant at *p* < 0.05 for experiments conducted in technical triplicate.

Differences were considered significant at *p* < 0.05. For morphometric image analyses, non‐parametric statistics were applied. Specifically, a Kruskal–Wallis test (*p* < 0.05) followed by Dunn's post hoc test with Holm correction was performed.

## 3 Results

### 3.1 In Vitro Effects of EDCs and RACs on SH‐SY5Y Cell Morphology

**FIGURE 1:**
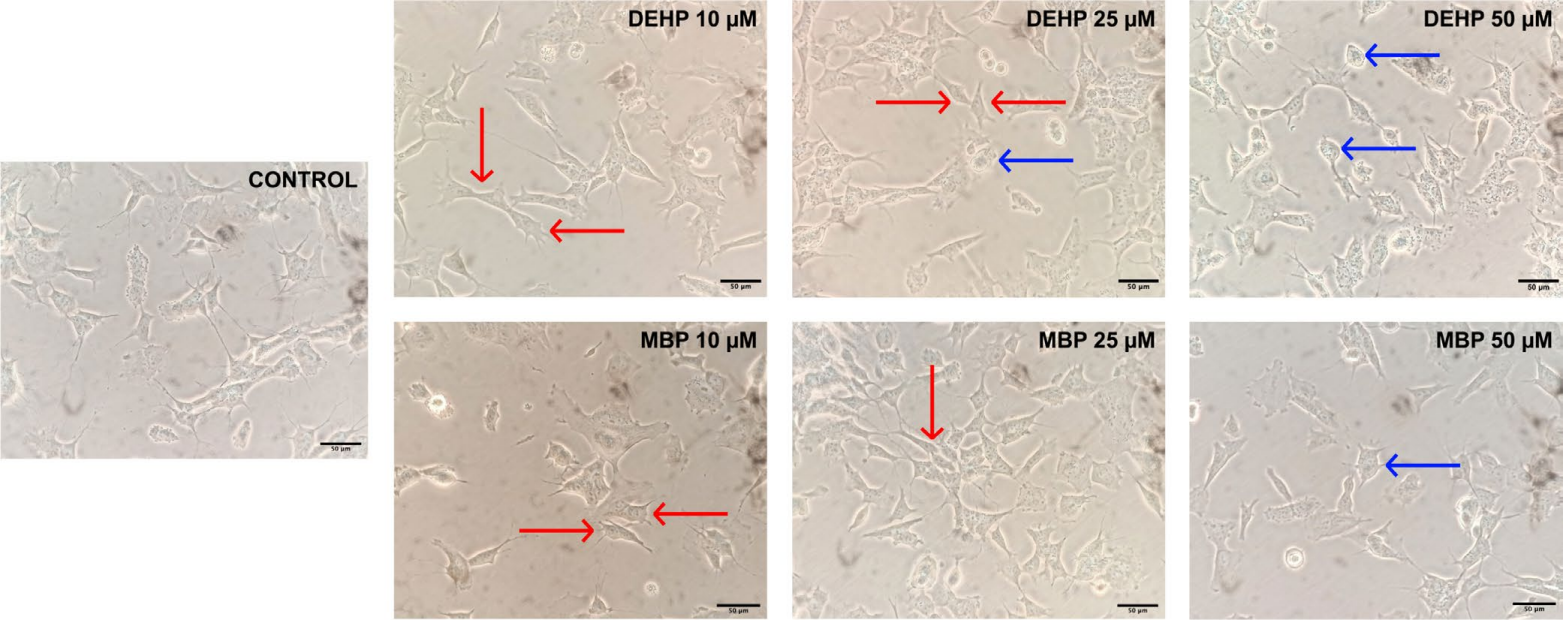
Effects of 24 h treatment with EDCs (DEHP and MBP) on the in vitro of SH‐SY5Y neuroblastoma cells. Representative photographs observed with a 40× objective. Scale bar 50 μM. From left to right: Control, followed by DEHP and MBP treatments at 10, 25, and 50 μM. Arrows indicate the cellular phenotype: Red for neuroblastic cells (N) and blue for substrate‐adherent cells (S).

**FIGURE 2:**
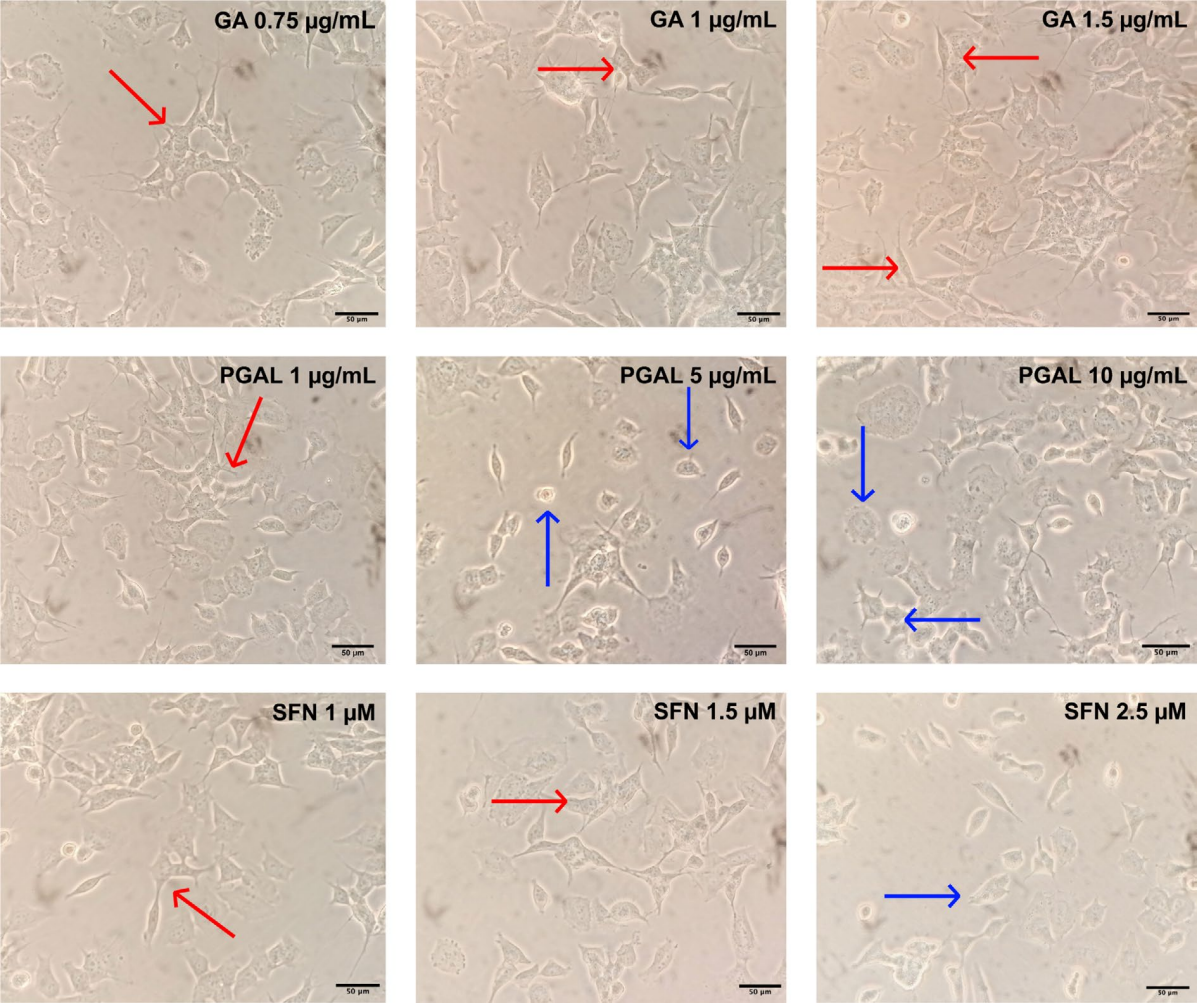
Effects of 24 h treatment with redox‐regulating molecules on the in vitro morphology of SH‐SY5Y neuroblastoma cells is depicted. Representative photographs observed with a 40× objective. Scale bar 50 μM. From left to right: Control, followed by treatment with GA 0.75, 1, 1.5 μg/mL, PGAL 1, 5, and 10 μg/mL, and SFN 1, 1.5, and 2.5 μM. Arrows indicate the cellular phenotype: Red for neuroblastic cells (N) and blue for substrate‐adherent cells (S).

Cell micrographs with a 40× for 24 h standard SY5Y cell culture are shown in Figure 1 displaying constant cell density and a characteristic neuroblastic (N) phenotype. Differences in cell density are not found when cells are treated with DEPH and MBP, but on the cells' phenotype. When treated with these EDC at 10 μM, they remain homologous to the control. Nevertheless, cells adherent to the substrate (S) are found when treated with 25 and 50 μM [23] (Figure 1). On the other hand, the comparison between cells treated with RACs and the control shows a variation in cell density, besides diminished cell viability with SFN in 2.5 μM concentration. There are no notable changes in the samples' phenotype when treated with GA 0.75, 1, 1.5 μg/mL; PGAL 1 μg/mL and SFN 1, 1.5 μM, compared to the control. However, the presence of cells adherent to the S is noted when increasing the polymer concentration to 5, 10 μg/mL, and also with SFN 2.5 μM (Figure 2).

**FIGURE 3:**
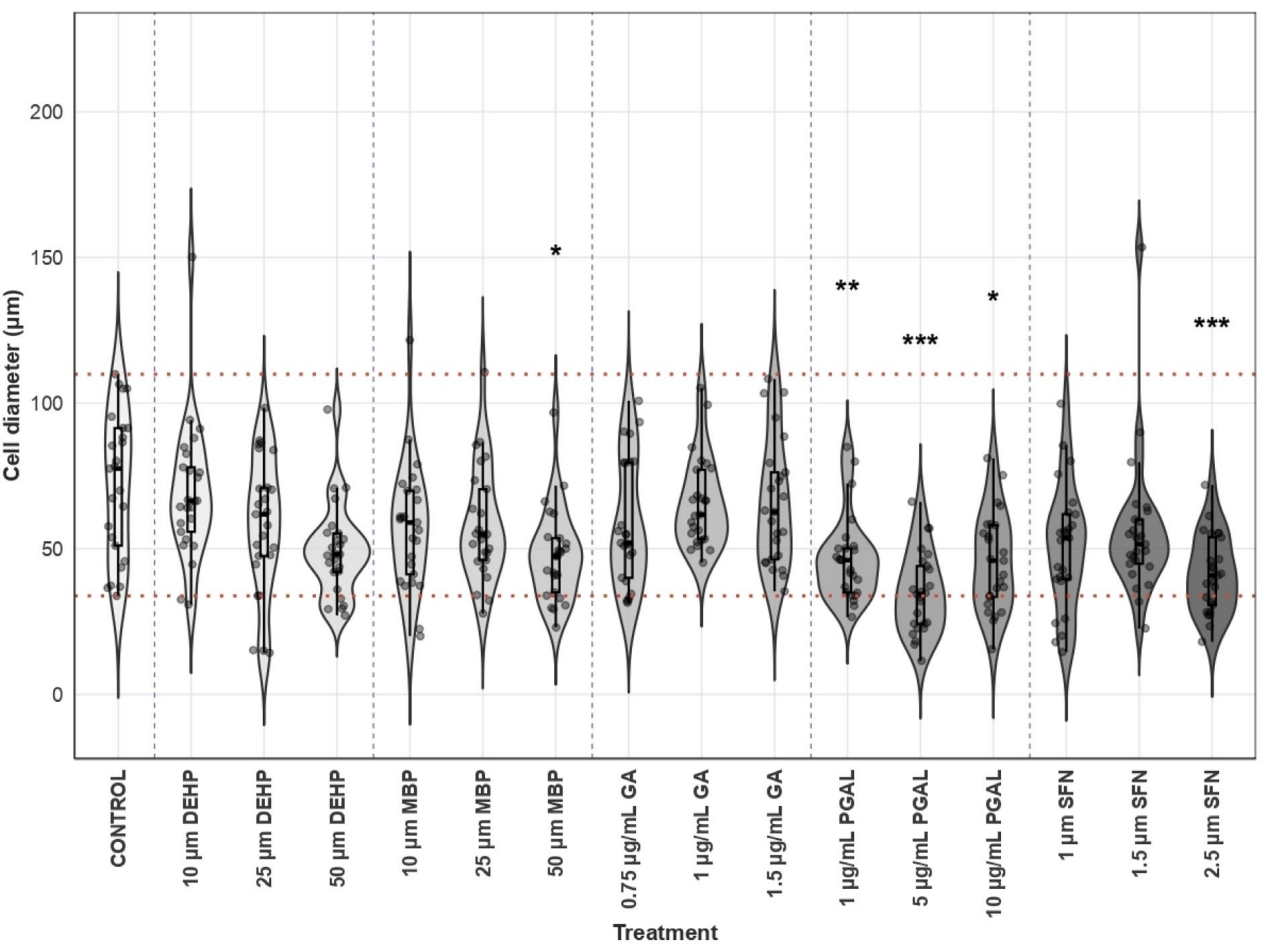
Morphometric analysis of cell diameter in SH‐SY5Y under different experimental conditions of EDC and RACs. Cell diameter was evaluated (*n* = 25 measurements per condition; total = 400 observations) using a Kruskal–Wallis test (*p* < 0.05), followed by Dunn's post hoc analysis with Holm's correction. The violin plots show the full data distribution and the median for each treatment. The red dashed horizontal lines indicate the characteristic range of cell diameter under control conditions (≈70–110 μm) and the blue dashed vertical lines group the different treatments evaluated. Treatments with 1.5 μg/mL GA, 10 μg/mL PGAL, 1 μM SFN and 2.5 μM SFN presented a significant reduction in cell diameter (*p < 0.05, **p < 0.01, or ***p < 0.001).

In order to further analyze morphological shifts in SH‐SY5Y neuroblastoma cells, we performed a statistical analysis of cell diameter (Figure 3). The results show that treatment with either 1.5 μg/mL GA, 10 μg/mL PGAL, 1 μM SFN, and 2.5 μM SFN resulted in statistically significant reductions in cell diameter.

### 3.2 In Vitro Effect of Exposure to EDCs and RACs on SH‐SY5Y Cell Viability

The cell viability of EDC and RAC treated samples displays a significant 34% increase for DEPH at the concentrations of 25 and 50 μM. Contrary, we have a 20% cell viability decrease with DEPH 1 μM. For the supposed RAC, we have a 12% and 20% decrease with GA 0.75 and 1 μg/mL, respectively, and a similar trend by 14% and 42% decrease when treated with SFN in 2.5 and 5 μM concentrations, respectively.

### 3.3 In Vitro Effect of Exposure to EDC and RAC on the Enzymatic Activity of MS on SH‐SY5Y Cultures

The MS specific activity increases 64% in the presence of DEHP 25 μM compared to control. This increase is 49% and 18% with MBP at 10 and 50 μM treatments, respectively. For GA, there are increases of 24% and 35% at 1 and 1.5 μg/mL concentrations compared to control, respectively, and raises to 37% with SFN 2.5 μM.

Remarkably, the MS enzymatic specific activity decreased 52%, 57%, and 38% with PGAL 1, 5, and 10 μg/mL, respectively. A decrease is also measured with SFN but at 1.5 μM concentration (45%) (Figure 5).

### 3.4 In Silico Prediction

In silico prediction displays that protein–ligand interactions occur in the vicinity of the folate‐binding domain for DEHP and PGAL and at the homocysteine‐binding domain for MBP, GA, and SFN, respectively. Interestingly, a loop‐like PGAL conformer can also dock at the interphase between the two catalytic domains (Figure 6).

## 4 Discussion

### 4.1 In Vitro Effects of EDCs and RACs on SH‐SY5Y Cell Morphology

Figures 1 and 2 show representative micrographs of cells under the experimental conditions conducted in the present work after 24 h exposure. For control experiments, the cell density remains constant throughout the treatment, displaying a slightly elongated fibroblast‐like phenotype with a median cell length of 77.49 μm. Cell contours were defined and lightly angular, from which neurites emerge, which is a typical characteristic of the N phenotype for SH‐SY5Y neuroblastoma. This agrees with findings from a detailed in vitro characterization of the SH‐SY5Y neuroblastoma cell line (ATCC (CRL‐2266)) under standard conditions [23]. The SH‐SY5Y neuroblastoma cell culture grows forming clusters of cells with multiple thin and short cellular extensions called neurites or neuronal processes (ATCC). Additionally, these types of cultures may present neuroblastic cells (N) and substrate‐adherent cells (S) phenotypes [44, 45].

Although not all treatments showed significant differences compared to control experiments, changes in cell diameter were evidenced. As can be seen in Figure 3, morphological shifts were identified in addition to the qualitative evaluation of the micrographs. The quantitative analysis objectively supports the morphological variations induced by the treatments and strengthens the overall interpretation of the results presented herein.

In this regard, cell cultures treated with EDCs and redox‐regulating molecules display variations in density and phenotype depending on their concentrations. The presence of DEHP and MBP at 10 and 25 μM shows a similar pattern for both treatments with median cell lengths ranging from 54.93 to 66.35 μm (Figure 3). Specifically, the cell density decreases minimally with a predominant N phenotype. However, the contours are angular, and in some cases visibly rounded, reminiscent of epithelial cells, which is the characteristic S phenotype for [23].

On the other hand, the predominant cellular morphology in the presence of DEHP and MBP at 50 μM is the S phenotype. As can be seen in Figure 3, the median cell length for DEHP 50 μM was 48.12 μm, while MBP 50 μM showed a median cell length of 47.43 μm, the latter displaying a statistically significant reduction compared with the control (*p < 0.05). Noteworthily, MBP treatment induces a significant decrease in cell density. Various studies have shown that exposure to phthalates is related to proliferation and cellular phenotypic modifications. In agreement with our study, A549 fibroblast cells challenged with DEHP and mono‐(2‐ethylhexyl) phthalate (MEHP), a DEHP metabolite, presented cell enlargement and elongation [46]. However, other authors found an increase in size and neurite production in PC12 cells exposed to MEHP at concentrations ranging from 2.5 to 250 μM, which disagrees with our results [47].

Exposure to GA (0.75, 1, and 1.5 μg/mL) resulted in a moderate decrease in cell density (see Figure 3), with a predominant N phenotype. Cells treated exhibited median cell lengths between 51.94 and 62.59 μm and displayed short bipolar fusiform cells, suggesting reduced cytoplasmic volume potentially associated with cell‐cycle alterations or apoptosis. This aligns with findings in HepG2 cells, where GA reduced confluence, induced rounding, and triggered apoptosis [48] (Figure 2).

We next evaluated the effects of the PGAL polymer on cultured SH‐SY5Y neuroblastoma cells. Across all concentrations tested, PGAL induced a minimal reduction in cell density; however, the cells appeared less elongated and predominantly displayed an S‐type phenotype, characterized by angular yet rounded contours and short neurites. Consistent with these morphological features, all PGAL treatments produced significant reductions in median cell length compared with the control. The most pronounced effect was observed at 5 μg/mL, with a median length of 34.33 μm and a highly significant difference (***p < 0.001), where cells exhibited a compact, short bipolar spindle‐like morphology (see Figure 3). At 1 μg/mL, the median length was 46.07 μm with a significant reduction (**p < 0.01), and at 10 μg/mL, the median length was 45.91 μm with a moderate but significant decrease (*p < 0.05). Together, these findings indicate a concentration‐dependent shift toward a less differentiated, substrate‐adherent phenotype (Figure 2). This is consistent with earlier works with PGAL using THP‐1 cells and fibroblasts showing changes in cell density and phenotype. A rounded phenotype has been reported for unstimulated THP‐1 cells, where PGAL inhibited cell adhesion. On the other hand, a typical fascicular phenotype is reported in fibroblast cells treated with PGAL at concentrations of 100 and 200 μg/mL [26, 49]. Similarly, cell viability in the presence of this polymer has been recently demonstrated in primary synoviocyte cultures with potential applications in arthritis [28].

Finally, treatment with SFN (1 and 1.5 μM) minimally decreases cell density, while inducing an N phenotype, with median lengths between 53.73–51.57 μm. In contrast, 2.5 μM SFN markedly reduced density and cell length, yielding a median of 40.81 μm, as well as the observation of increasing rounded cells with few or no neurites.

Finally, SFN treatment at 1 and 1.5 μM minimally decreased cell density while inducing an N phenotype, with a median cell length between 53.73 and 51.57 μm. In contrast, 2.5 μM SFN markedly reduced both cell density and cell length, resulting in a significantly shorter median length of 40.81 μm (**p < 0.01), along with an increased presence of rounded cells showing few or no neurites (see Figure 3). This is consistent with a potential hormesis, in which a drug or molecule at low concentrations can have protective effects, while at high concentrations, the same compound can have an inhibitory effect depending on its concentration or cell type [50]. For example, a reduction in oxidative stress has been reported in shFxn upon addition of 5 μM SFN with a greater number of neurites, potentially associated with increased brain plasticity [51]. Additionally, co‐culture of N9 cells activated with lipopolysaccharide (LPS) results in a significant increase in SH‐SY5Y cell death but a significant decrease in neurite length [52]. Pre‐treating these same cultures with 5 μM SFN results in a protective effect where neurite length increased. This suggests that pre‐treatment with SFN could significantly reduce the neurotoxic effects of activated microglial cells. It is noteworthy that neurite modification is related to a complex interaction of genetic, environmental, cellular signaling, cell–cell interactions, and neuronal architecture factors. Therefore, the study of these interrelationships is crucial to understanding the formation, function, and protection of the nervous system.

### 4.2 In Vitro Effect of Exposure to EDCs and RACs on SH‐SY5Y Cell Viability

Phthalates represent one of the most relevant contaminants in food due to their ability to migrate from plasticized materials and act as endocrine disruptors [11, 53]. Within this group, two compounds with contrasting but complementary profiles were chosen.

DEHP is one of the most frequently detected phthalates in food matrices. It has very low aqueous solubility (~0.3 mg/L), high affinity for organic solvents and lipids, and a melting point of −50°C, characteristics that explain its marked lipophilicity and its preferential accumulation in cell membranes and fatty tissues [54].

MBP, in contrast, is the most bioactive and toxic monoester metabolite of DBP (dibutyl phthalate). It exhibits moderate solubility in water, high solubility in organic media, and a melting point of −15°C. Its higher water solubility favors its rapid distribution in aqueous compartments and excretion, although it retains sufficient lipophilicity to interact with cellular structures [55].

The comparison between the two compounds is relevant to the study, since it allows us to evaluate the response in neuronal metabolic activity against two phthalates with different physicochemical properties and biodistribution patterns: a highly lipophilic diester (DEHP) and a water‐soluble monoester (MBP), recognized for its higher toxic activity.

**FIGURE 4:**
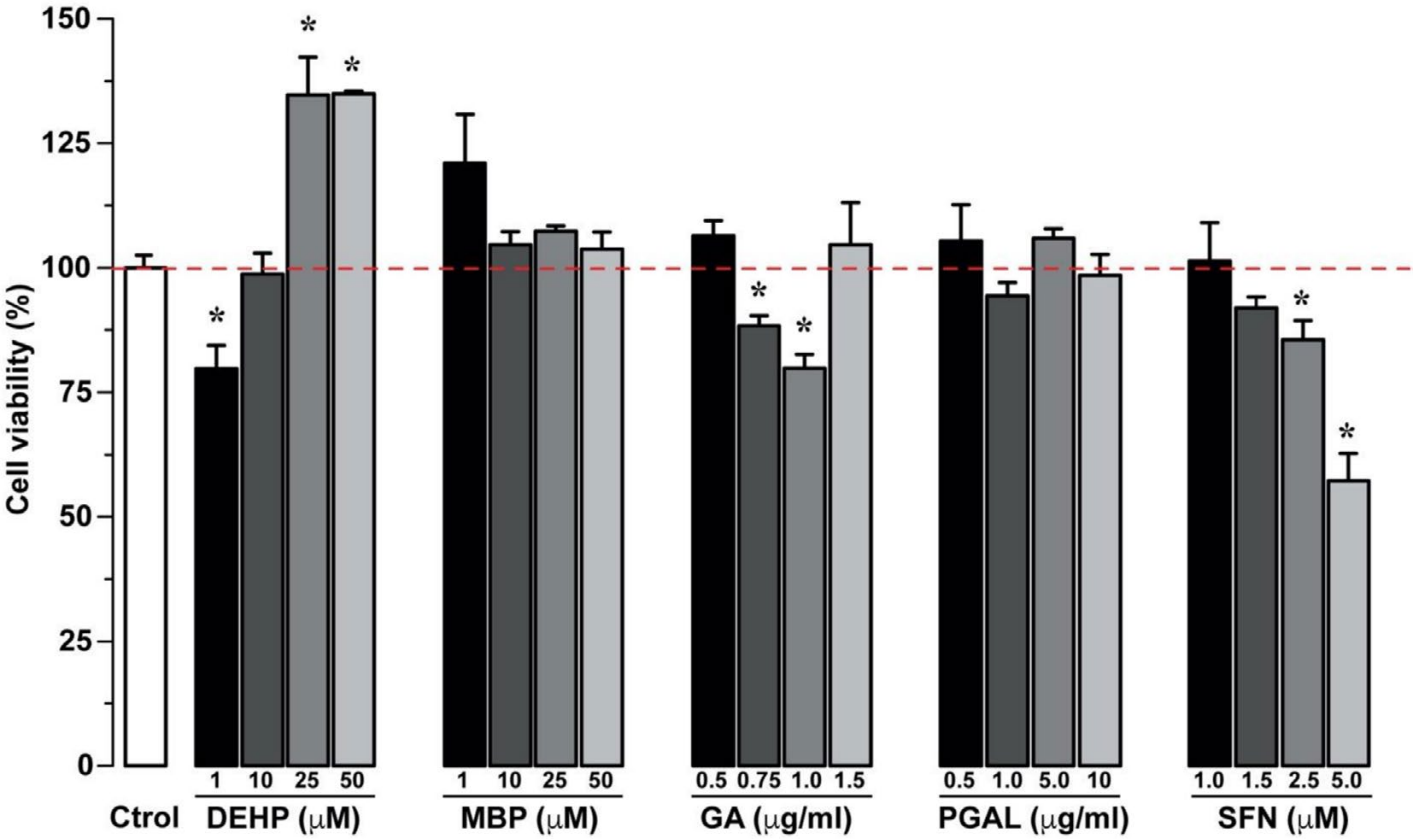
In vitro Cytotoxicity of EDC and redox regulatory molecules at different concentrations in SH‐SY5Y neuroblastoma cultures after 24 h treatments. Percentage graphs relative to control. The red line represents the control average. Data are presented as mean ± SD of triplicate experiments. *p* < 0.05 versus control.

The analysis conducted over a 24 h period confirmed that exposure to 1 μM DEHP induces a dose‐dependent reduction in cell viability (79.78%, ±7.98) compared to control. In contrast, viability remained either unaffected or increased with 10, 25, and 50 μM DEHP (98.76%, ±7.24; 134.65%, ±13.21; 134.95%, ±0.92) (see Figure 4). The cytotoxicity for DEHP has been evaluated in both human (SH‐SY5Y) and mouse (Neuro‐2a) neuroblastoma cell lines. Previous findings report an initial increase in viability at lower DEHP concentrations (0.1 μM), followed by a significant decrease in higher concentrations (10, 50, and 100 μM) for SH‐SY5Y [13]. For the Neuro‐2a line, on the other hand, it has been found that viability remains constant or even increases slightly at low DEHP concentrations (1 μM). In the present work, a significant decrease is observed starting at 10 μM of DEHP and followed by a further decrease at 100 μM [56, 57].

The significant increase in cell viability due to EDCs may not be a signal of the increase in cellular production but a consequence of different reactions in cellular metabolism, where enzymes related to the homeostatic deregulation caused by the EDCs are activated. This agrees with a study conducted with MDBK cells treated with tert‐butylhydroquinone [58]. This commercial antioxidant activated the enzyme glucose‐6‐phosphate dehydrogenase and generated NADPH, which in turn reduced the XTT in the medium culture.

In this study, we also notice that exposure of cell cultures to 1 μM MBP results in a significant increase in viability (121.02%, ±17.04) compared to control. Additionally, viability remains constant at concentrations of 10, 25, and 50 μM (104.67%, ±4.44; 107.35%, ±1.79; and 103.75%, ±5.89, respectively) (Figure 4). There are few studies on the cytotoxicity caused by MBP; however, its adverse effects at high concentrations on Sertoli and MLTC‐1 cells have been previously described [59]. In the case of the MLTC‐1 cell line, a similar effect has been reported, with viability remaining constant at concentrations up to 100 μM but showing a significant decrease at 1000 and 2000 μM for a 24 h period [47].

On the RAC side, the GA and SFN are widely studied molecules with neuroprotective effects, as their use is reported for the treatment of free radical‐induced disorders of the nervous system [60, 61], since redox‐regulating molecules are typically involved in the defense system against ROS. However, it is very important to pay attention that RAC levels depend on the model and cell line. For example, a cytotoxic effect has been observed at concentrations higher than 50 μg/mL of GA and 10 μM of SFN [30]. According to this, we assess the 0.5, 0.75, 1, and 1.5 μg/mL for GA concentrations (see Figure 4) where we have a minimal but significant decrease in viability at concentrations from 0.75 to 1 μg/mL (88.34%, ±3.62 and 79.87%, ±4.72). In contrast, viability increases after 24 h at 1.5 μg/mL (104.67%, ±14.47). Reported works on GA suggest that this metabolite can suppress cell viability in a concentration‐dependent manner, that is, it prevents cell proliferation by inducing apoptosis at higher concentrations in cell lines, including U937 [62], A549 [63], and HeLa [64]. Another example is U87 and U251 glioma cells showing selective cytotoxicity depending on the cell type and GA concentration [65]. It is worth mentioning that GA is sparingly soluble in water, but at physiological pH adopts a soluble gallate structure, which is thermo and photo‐sensitive, and transforms into quinoid forms losing its antioxidant capacity. Therefore, different experimental factors and cell lines may influence their bioavailability, which is important to consider when assessing the dose–response. In the case of SFN, we have changes in cell viability at 1 and 1.5 μM (Figure 4). However, there is a significant decrease in cell viability at 2.5 μM SFN (85.60%, ±6.58), which markedly decreases at 5 μM (57%, ±9.42). This may be related to the cytotoxicity of SFN reported elsewhere for SH‐SY5Y neuroblastoma cells [35, 61] and Neuro‐2a [66].

On the other hand, the antioxidant and anti‐inflammatory properties for PGAL have been demonstrated in different cell lines including THP‐1, HCT 116, and HT‐29 among others, showing a significant decrease in proliferation and cell adhesion with treatments ranging between 100 and 200 μg/mL [26]. Additionally, no adverse effects on cell proliferation have been observed in dermal fibroblasts and primary synoviocytes (even at high concentrations) for this polymer [28, 49]. Considering the characteristics of the SH‐SY5Y neuroblastoma cell line, and in accordance with previous studies on PGAL, we study relatively low PGAL concentrations (0.5, 1, 5, and 10 μg/mL) to identify potential cytotoxic effects. The results shown in Figure 4 indicate a mild increase in cell viability after 24 h of treatment with 0.5 and 5 μg/mL (105.40%, ±12.63; 105.97%, ±3.24) and no changes in viability and adhesion as compared to control experiments for 1 and 10 μg/mL (94.44%, ±4.47 and 98.49%, ±7.21). Worth noting that cell adhesion is maintained in cultures when exposed to PGAL, notwithstanding the reduction in adhesion of fibroblasts previously reported at 100 and 200 μg/mL [26]. Since cell adhesion is advantageous for properly identifying multiple parameters including cytotoxicity and enzyme activity, and to ensure the absence of any cytotoxic effects, we keep low PGAL concentrations in this work.

### 4.3 In Vitro Effect of Exposure to EDC and RAC on the Enzymatic Activity of MS on SH‐SY5Y Cultures

Further study on the outcomes of DEHP and MBP treatments on enzymatic activity of MS is based on previous evidence showing that in the Neuro‐2a cell line significantly decreases its activity in the presence of these two EDCs in food products [51]. To our knowledge, this is one of the first studies identifying MS enzymatic activity in human neuroblastoma SH‐SY5Y cell line cultures, as a bona fide model to test EDC disruption in an in vitro context. The cells of male origin might be more sensitive to EDC due to the presence of androgen hormones, which could influence neurodevelopment [67]. There are no male neural cell lines; however, it has been recently reported the use of induced pluripotent stem cells (iPSC) to differentiate them into male neural cells [68], which could be used in future studies to expand the knowledge of the effects of EDCs in other male cells, as reported elsewhere [69].

The SH‐SY5Y cell line is of female origin and well‐recognized and characterized as a neuronal model widely used in neurobiological and toxicological research [70]. Its ability to differentiate into neuronal phenotypes allows us to assess the neurotoxic effects of emerging contaminants reliably. For example, this cell model has been reported to identify the epigenetic consequences of BPA exposure [24]. Furthermore, the use of female SH‐SY5Y cells enables inclusion of the perspective of EDC exposure during pregnancy since the mother–child binomial has been acknowledged as the most vulnerable population [71]. From a biochemical and cellular perspective, this pairing involves the maternal transfer of factors such as hormones, metabolites, and xenobiotics across the placenta and blood–brain barrier, which influence critical signaling pathways, specifically the interference due to nuclear receptor activations and protein modulation involved in oxidative stress and inflammation [72]. In this regard, MS activity is a core element involved in oxidative cellular processes and therefore a potentially key sensor to identify endocrine disruption by emerging contaminants.

**FIGURE 5:**
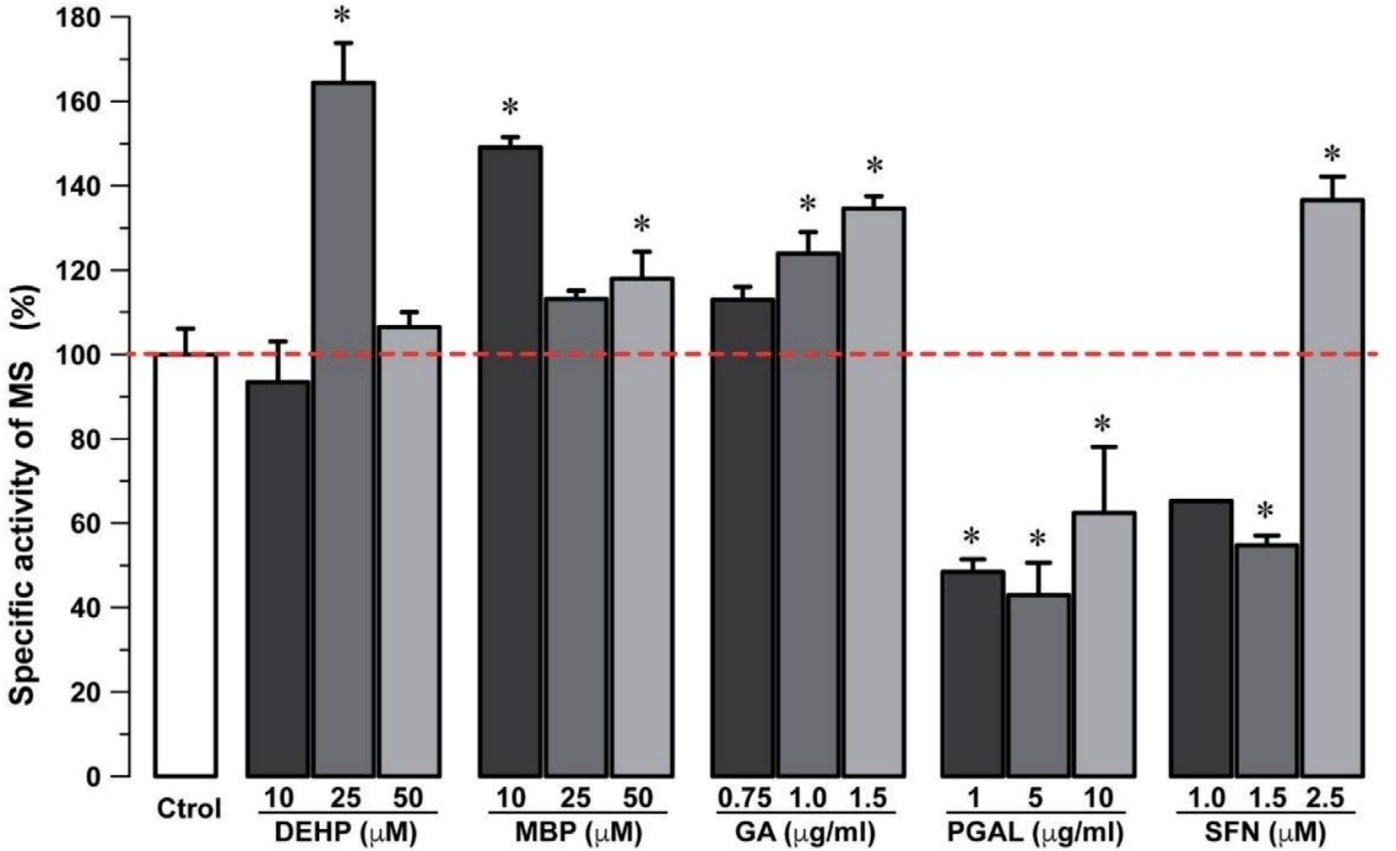
Specific enzymatic activity of MS in SH‐SY5Y neuroblastoma cultures at 24 h treatments. Two‐way ANOVA and post hoc comparison tests were conducted. Data are presented as mean ± SD of triplicate experiments. *p* < 0.05 versus control.

As shown in Figure 5, the monitoring of specific activity of MS after 24 h and the comparison of EDCs and RAC experiments with control provide interesting outcomes. First, there is a specific MS activity of 93.46% ± 16.60% for DEHP in 10 μM, indicating a slight reduction compared to control conditions. For 25 μM DEHP, however, there is a significant increase to 164.43% ± 16.43%, and for this EDC at 50 μM, a minimal increase in MS activity of 106.48% ± 7.12% (Figure 5). On the other hand, MBP induces the increase in specific MS activity, which is significant at 10 and 50 μM, with 149.17% ± 4.034% and 117.91% respectively, and minimal for 25 μM, with 113.18% ± 30.61%, 30.613% ± 0.922% (Figure 5).

Several authors have reported that DEHP induces oxidative stress, leading to neurotoxicity in mouse neural cells NE‐4C and HT22 [73, 74]. The explanation of the mechanism is still elusive; nevertheless, these studies identified significant variations in metabolites regulating cellular redox in the presence of 5 and 10 μM DEHP. For example, these authors report an increase in the levels of malondialdehyde (MDA), a decrease in glutathione (GSH) ratio, as well as in the activities of superoxide dismutase (SOD) and glutathione peroxidase (GSH‐PX). Our results indicate that EDCs collectively increase the specific activity of MS, and in some cases, this increase is significant, such as with DEHP (25 μM) and MBP (10 and 50 μM). Meanwhile, potentially redox‐regulating molecules display distinct patterns in MS activity; that is, a significant increase in GA (at 1 and 1.5 μg/mL) and SFN (at 2.5 μM) but a significant decrease in PGAL (at 1, 5, and 10 μg/mL) and SFN (at 1 and 1.5 μM).

As observed in Figure 5, non‐monotonic dose–response relationships between EDCs' concentrations and MS activity are observed. It is relevant to note that several EDCs, including phthalates and compounds of redox nature, have shown non‐monotonic behaviors, characterized by different or even opposite responses depending on the concentration [75, 76]. This phenomenon is widely documented in endocrine toxicology and is consistent with the type of variations observed in MS activity in our cell model.

DEHP is a molecule 1.75‐fold heavier than MBP, and DEHP is lipophilic in contrast to MBP, which is hydrophilic due to its COOH moiety [77]. Earlier reports indicated that DEHP lipophilicity enables it to passively diffuse through the cell membrane, leading to accumulation in membranes and organelles, which are enhanced at low concentrations. Besides, DEHP has been reported to modify membrane fluidity and therefore alter cellular processes without further biotransformation and trigger oxidative stress cell responses [78]. On the other hand, MBP has been observed to penetrate the cell membrane by facilitated diffusion or active transport due to its polarity, thereby increasing its probability of biological interaction. Specifically, low concentrations of MBP in zebrafish liver cells show no toxic effects and can even promote cell viability [79]. Nevertheless, ROS production is related to organelles such as mitochondria that are associated with apoptotic mechanisms. Thus, high concentrations of MBP can be ROS‐producing by interacting with different mechanisms [80].

In summary, the non‐monotonic effects observed in MS activity may be due to differential interaction of DEHP and MBP with concentration‐dependent cell signaling pathways [64].

The treatment with GA increases the specific activity of MS depending on the concentration used, from 112.98% ± 5.27% at 0.75 μg/mL to 123.93% ± 8.80% and 134.60% ± 5.11% at 1 and 1.5 μg/mL, respectively (Figure 5). These results contrast those with PGAL, which is capable of lowering this enzymatic activity to 48.45% ± 5.10% and 42.98% ± 13.29% at 1 and 5 μg/mL, respectively, with a slight increase to 62.40% ± 27.23% at 10 μg/mL. These results match others where PGAL treatments at 10, 100, and 200 μg/mL strongly reduce ROS production and cell death in fibroblast cells [49], which merits further work on the potential PGAL neuroprotective effects.

Similarly, SFN decreases MS‐specific activity at 1 and 1.5 μM, being 65.21% ± 0.41% and 54.76% ± 3.85%, respectively. A significant increase of 136.60% ± 9.59% is recorded at 2.5 μM (Figure 4). Notably, the effect of SFN as an antioxidant has been evaluated in SH‐SY5Y cells at 5 μM for 24 h before the administration of pyruvaldehyde at 500 μM for an additional 24 h [81]. That study concludes that SFN prevents mitochondrial redox impairment by stimulating the activity of the enzyme γ‐glutamyl cysteine ligase, leading to increased GSH synthesis.

Our findings show that SFN and PGAL can decrease MS activity and highlight the regulatory effect of proven RAC molecules on this enzyme. However, GA has opposite results on MS activity, although the reports on its neuroprotective action in different models of neurodegeneration, neurotoxicity, and oxidative stress [82, 83]. GA has been reported to play a protective role by improving cellular antioxidant status and inhibiting the production of proinflammatory cytokines in primary cultures of rat cortical neurons at 25 and 50 μg/mL [84]. Nonetheless, the activity expected for MS under GA treatment does not match those for the other two RACs studied in this paper, and further work is needed to disclose the mechanism involved.

**FIGURE 6:**
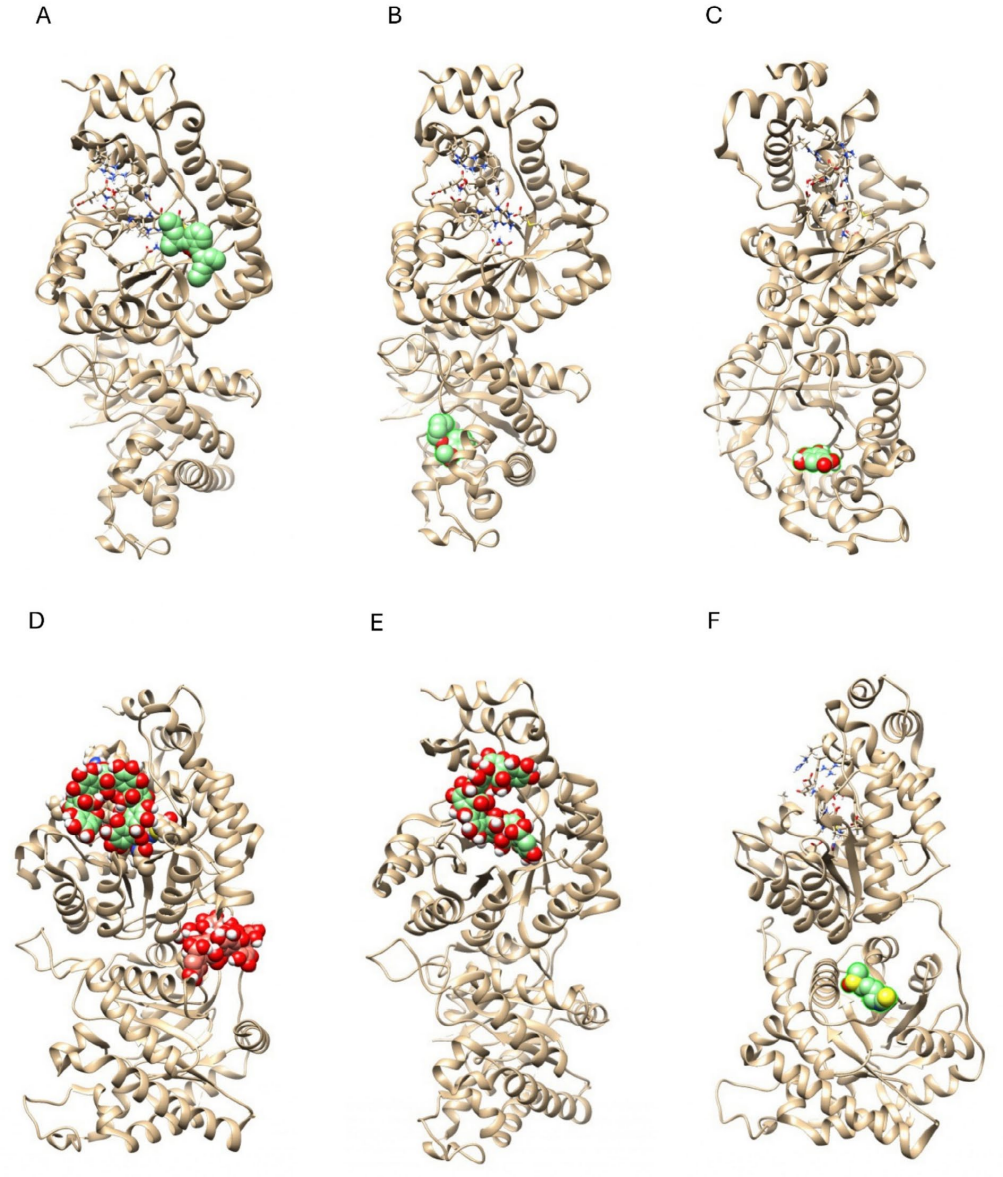
Molecular docking of MS with endocrine disruptors. Ligands were docked to the surface of human methionine synthase (MS) (PDB: 4CCZ) as predicted by AutoDock Vina. Protein conformations are shown as ribbon models, tetrahydrofolate (THF) is shown in stick representation, and tested ligands are depicted in ball representation. The binding pose with the lowest docking score and RMSD is shown for (A) DEHP, (B) MBP, (C) GA, (D) PGAL, (E) PGAL bound to MS in the absence of cofactor THF, and (F) SFN. Protein–ligand interactions are predicted to occur close to the folate‐binding domain for DEHP and PGAL and at the homocysteine‐binding domain for MBP, GA, and SFN, respectively. PGAL can also dock to the interphase between both catalytic domains. Molecular models were generated using Chimera and were rotated to show each ligand.

As shown in Figure 6, an in silico molecular docking model in human MS predicts that DEHP binds near the folate‐binding domain of the protein, while MBP, GA, and SFN exhibit the highest docking probability near the homocysteine‐binding domain. Based on our findings, we hypothesize that DEHP binding near the folate‐binding domain may stabilize the active site, potentially favoring the conversion of homocysteine to methionine. This, in turn, would increase the availability of methyl groups for various methylation reactions, including dopamine‐stimulated phospholipid and DNA methylation [85]. In contrast, MBP and GA interactions within the homocysteine‐binding domain may regulate substrate accessibility, thereby promoting catalysis [86]. Conversely, SFN binding in this same domain could induce conformational changes that decrease methionine synthase activity. Interestingly, PGAL binds in two potential pockets: one located exactly over the folate‐binding domain and the other one at the interphase between the two domains of MS. While the former would explain the dramatic decrease in MS specific activity, the latter may also impair catalysis by uncoupling both domains [18]. It is important to note that the conformations shown in the docking snapshots (Figure 6D,E) represent local interactions between a short PGAL segment and the enzyme surface. These snapshots should not be interpreted as global models of the full polymer architecture. In extended PGAL chains (e.g., a 16‐unit model; Figure S1), low‐energy conformations predominantly adopt open or coil‐like geometries, and loop‐like arrangements analogous to those in Figure 6D,E do not arise spontaneously. Thus, the apparent ‘loop’ observed in the docking result reflects a local wrapping of a flexible polymer fragment, rather than a stable circular conformation of the entire PGAL chain.

Our results indicate that in the presence of contaminants and reactive species, MS activity increases by 5%–30% compared to the control. In contrast, activity decreases in the presence of two recognized redox regulators, PGAL and SFN. Notably, at 2.5 μM SFN—a concentration that is not cytotoxic but has been reported to induce cellular stress in some cases—MS activity is not significantly reduced, notwithstanding the reported decrease in cell viability in Neuro2a neuroblastoma cells at high SFN concentrations [66]. Remarkably, the polymer PGAL is herein demonstrated as a potential redox regulator and preserver of neuritic cells.

MS activity is essential for homocysteine‐ and folate‐dependent methylation of phospholipids stimulated by the dopamine D4 receptor, a unique signaling process that promotes the synchronization of neural networks [18]. Dysregulation in the metabolism of MS activity by inhibition leads to alterations in DNA methylation, which have been associated with behavioral and cognitive alterations, mainly in the following areas [87]. Today, the physiologically significant ranges are highly variable and depend on the epigenome of every individual and region [88]. Consequently, further work is in progress to deepen the mechanisms of action of EDC exposure to dysregulate MS and to evaluate the potential use of RACs as neuroprotective molecules.

## 5 Conclusions

PGAL modulates MS activity, and this enzyme‐mediated polymer is a potential RAC for SH‐SY5Y neuroblastoma cells. Our study shows that molecules can cause cytotoxicity at concentrations of 10 μM for DEHP, 0.75 and 1 μg/mL for GA, and 2.5 and 5 μM for SFN, but no toxicity for any PGAL concentrations. Furthermore, MS activity is significantly increased for EDCs at 25 μM DEHP, as well as in 10 and 50 μM of MBP. A significant increase is also observed in the presence of 1 and 1.5 μg/mL of GA, as well as with 2.5 μM of SFN. On the other hand, a significant decrease is recorded at 1, 5, and 10 μg/mL of PGAL, and 1 and 1.5 μM for SFN. It is noteworthy to mention that cumulative exposure to EDCs can take place through food ingestion, potentially altering cellular redox homeostasis. This alteration has been associated with changes in DNA redox metabolism. Thus, quantitative evaluation through specific biomarkers, such as MS, represents a novel proposal that paves the way for future research.

To sum up, cumulative exposure to EDCs, particularly through dietary sources, may alter cellular redox homeostasis, a phenomenon linked to changes in DNA redox metabolism. Thus, quantitative evaluation of specific biomarkers such as MS represents a promising approach for elucidating the cellular impact of these compounds. In this context, the experimental evaluation of compound–MS interactions using recombinant proteins may constitute a key perspective for future work.

## Author Contributions

All authors contributed to the study conception and design. Material preparation, data collection, and analysis were performed by G.G.‐C. and R.G.‐A. The first draft of the manuscript was written by G.G.‐C., and all authors commented on previous versions of the manuscript. All authors read and approved the final manuscript. Conceptualization: R.G.‐A. Methodology: G.G.‐C., R.G.‐A., M.G.‐A. Formal analysis and investigation: Y.R.‐C., M.R.S., R.G.‐A., G.G.‐C. Experimental feedback and methodology insights: M.R.S. Writing review and editing: M.G., M.G.‐A., Y.R.‐C., M.R.S., I.H.B.‐C., F.S.‐B. Funding acquisition: R.G.‐A., G.G.‐C.

## Funding

This work was supported by Dirección General de Asuntos del Personal Académico, Universidad Nacional Autónoma de México, IT200824, Universidad Nacional Autónoma de México, PAIP 5000‐9154, and SECIHTI (CVU 1103731, CVU 1099898).

## Disclosure

The programs used are as follow: Plots were performed in OriginLab. Photos were taken in an optical microscope with a 40× objective, the scale bar was added in ImageJ, edited in InDesign, and exported in Photoshop. RStudio was used for the statistical analysis and related graphics. Chimera and AutoDock Vina were used for docking studies.

## Ethics Statement

The authors have nothing to report.

## Consent

All authors have read and approved the final manuscript and consent to its publication.

## Conflicts of Interest

The authors declare no conflicts of interest.

## Supporting information


**Figure S1:** Comparative low‐energy conformations of PGAL8 (A) and PGAL16 (B), generated using ETKDGv3 followed by UFF minimization. For each oligomer, an extended conformation (“open”, cyan), an intermediate coil (yellow), and a compact coil (magenta) are shown. Both chain lengths prefer coil‐like conformations with no evidence of stable looped geometry.

## Data Availability

The data that support the findings of this study are available from the corresponding author upon reasonable request.
