# Supplementary material for: Enzymatically Synthesized Poly(Gallic Acid) Modulates Methionine Synthase Activity and Neuroblastoma Morphology in Contrast to Phthalate‐Type Endocrine Disruptors
Source: Biopolymers. 2025 Dec 11;117(1):e70074. doi: 10.1002/bip.70074 (PMC12696780; doi:10.1002/bip.70074)
Supplement: Supplementary file 1 — Figure S1: Comparative low‐energy conformations of PGAL8 (A) and PGAL16 (B), generated using ETKDGv3 followed by UFF minimization. For each oligomer, an extended conformation (“open”, cyan), an intermediate coil (yellow), and a compact coil (magenta) are shown. Both chain lengths prefer coil‐like conformations with no evidence of stable looped geometry. [file BIP-117-e70074-s001.docx]

**Supplementary Figure.**

**
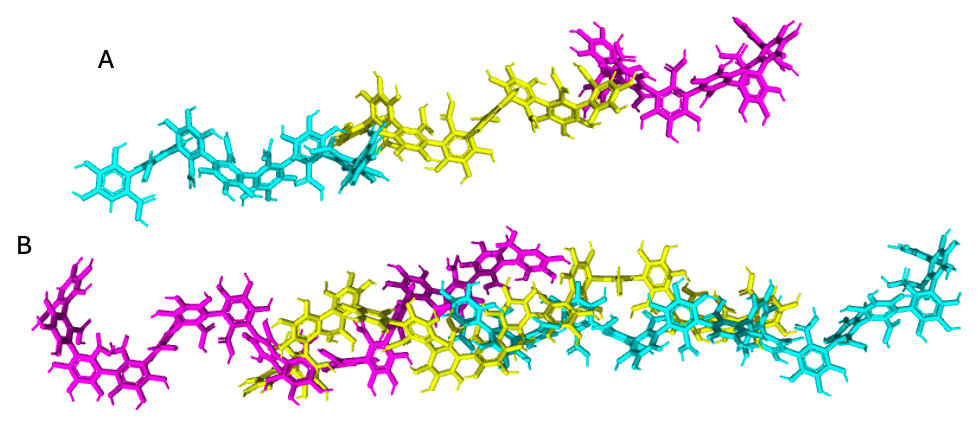
**

**Figure S1.** Comparative low-energy conformations of PGAL8 (A) and PGAL16 (B), generated using ETKDGv3 followed by UFF minimization. For each oligomer, an extended conformation (“open”, cyan), an intermediate coil (yellow), and a compact coil (magenta) are shown. Both chain lengths prefer coil-like conformations with no evidence of stable looped geometry.
